# Optimizing Airway Function Through Craniofacial and Cervical Manipulations and Emergency-Anesthesia Maneuvers: Applications in Airway Function Enhancement, Pneumonia, and Asthma—Narrative Review

**DOI:** 10.3390/jcm14134494

**Published:** 2025-06-25

**Authors:** Jason Park, Luz Benitez, Amethyst Hamanaka, Ghulam Husain Abbas, Emmanuel Faluade, Sjaak Pouwels, Jamie Eller

**Affiliations:** 1 College of Osteopathic Medicine, Sam Houston State University, Conroe, TX 77304, USA; lmb129@shsu.edu (L.B.); afh023@shsu.edu (A.H.); 2 Faculty of Medicine, Ala-Too International University, Bishkek 720048, Kyrgyzstan; husainghulam.162@gmail.com; 3 Department of Medicine, Mass General Brigham, Boston, MA 02114, USA; 4 Independent Scholar of Anesthesiology & Pain Medicine, Houston, TX 77004, USA; emmanuelfaluade@gmail.com; 5 Department of Surgery, Bielefeld University—Campus Detmold, Klinikum Lippe, 32756 Detmold, Germany; sjaakpwls@gmail.com; 6 Department of Intensive Care Medicine, Elisabeth-Tweesteden Hospital, 5022 GC Tilburg, The Netherlands

**Keywords:** osteopathic manipulative treatment, airway management, asthma, pneumonia, craniosacral therapy, cervical manipulation, lymphatic techniques, Chapman’s reflexes

## Abstract

**Background:** Even with advanced management involving pharmacologic and ventilatory strategies, respiratory dysfunction increases morbidity and reduces the quality of life. This narrative review examines how craniofacial and cervical manipulative interventions—including nasomaxillary skeletal expansion, breathing re-education, and structural techniques—may holistically optimize airway function by enhancing neurological and lymphatic dynamics, modulating vagal tone, reducing pharyngeal collapsibility, and supporting immune regulation across diverse clinical settings. **Objectives:** To explore manual techniques that influence respiratory and autonomic function and to evaluate their reported clinical efficacy and supporting evidence, particularly in the context of airway disorders such as asthma and pneumonia. **Methods:** A narrative review of the literature from PubMed and Google Scholar was conducted using search terms related to airway function and osteopathic manipulative techniques (OMTs). The inclusion criteria spanned 2010–2025 English-language peer-reviewed full-text articles on airway function, OMT, and emergency airway maneuvers. Clinical trials, observational studies, and reviews were included; non-peer-reviewed content and animal studies (unless mechanistically relevant) were excluded. Chapman’s reflexes related to respiratory function were incorporated to highlight somatic–visceral correlations. **Key Findings:** The techniques reviewed included frontal lift, vomer manipulation, maxillary and zygomatic balancing, and cervical adjustments. Thoracic OMT methods, such as diaphragm doming and lymphatic pump techniques, were also addressed. Emergency techniques, such as the BURP and Larson maneuvers, prone positioning, and high-frequency chest wall oscillation, were presented as comparative strategies to OMTs for acute airway management. **Conclusions:** Craniofacial and cervical manipulations can be a promising adjunct for enhancing airway function. However, the current literature displays heterogeneity and lack of large-scale randomized trials, which emphasize the necessity for standardized research and the establishment of clinical guidelines with the collected evidence.

## 1. Introduction

The presence of respiratory dysfunction and conditions such as pneumonia and asthma leads to increased morbidity rates and reduced quality of life (QoL) [1,2]. Craniofacial and cervical manipulative interventions, as part of holistic techniques, have been proposed to support respiratory health by influencing structural changes, neurological pathways, and lymphatic flow [3,4,5]. Scientific studies show that procedures involving nasomaxillary skeletal expansion along with breathing re-education help improve nasal airflow and pulmonary function while reducing pharyngeal collapsibility and chronic pain [6,7]. The adjustments leverage the interconnectedness of craniofacial structures, cervical spine, and airway functions to advance respiratory mechanics and improve immune responses and autonomic regulation. Additionally, Chapman’s reflexes (CRs)—specific neurolymphatic tender points—demonstrate predictable somatic–visceral relationships, as described in osteopathic educational resources [8]. For instance, anterior Chapman’s points for the lungs at third and fourth intercostal spaces (ICS) and posterior points at third and fourth thoracic transverse processes (T3 and T4) correspond to pulmonary health [8]. Taken together, the structural, autonomic, and somatic–visceral insights—such as CR mapping—provide a mechanistic rationale for using manipulative techniques to influence respiratory function. The review discusses the various craniofacial and cervical manipulation techniques that support optimized breathing and respiratory disorder management through their effects on structural alignment, vagal tone modulation, lymphatic drainage enhancement, and reduction in airway resistance. These interventions create a solid basis for complete respiratory care and symptom control by resolving particular dysfunctions in patients throughout various clinical settings. Moreover, it explores the role of craniofacial and cervical manipulations, as well as emergency airway maneuvers, in enhancing respiratory function in patients with asthma, pneumonia, and other airway dysfunctions. This review addresses a critical gap in the literature by synthesizing manipulative techniques that may influence airway and autonomic function in different clinical presentations, highlighting manual therapies—including osteopathic techniques—while noting that pharmacologic and mechanical ventilation strategies continue to dominate the current clinical approaches. By summarizing the mechanisms, clinical applications, and emergency airway alternatives, this review provides clinicians with a framework to understand how integrative manual techniques may serve as complementary strategies in respiratory care, particularly in patients who may benefit from non-pharmacological interventions.

## 2. Methods

This narrative review gathers collective information from peer-reviewed journal articles in clinical medicine on the subject of OMT as practiced and studied by physicians trained in osteopathic medicine (DOs), pulmonary physiology, and manual therapy in a flexible and practical manner appropriate for narrative analysis [9]. In addition to peer-reviewed literature from 2010 to 2025, we included key educational resources commonly referenced in osteopathic training—such as *The Cranial Bowl* by Sutherland (1939) [10] and clinically oriented summaries from StatPearls [8]—to contextualize foundational concepts like the primary respiratory mechanism (PRM), CRs, and associated manipulative techniques. A thorough database search was conducted using PubMed and Google Scholar. The search terms included “airway function”, “osteopathic manipulation”, “frontal lift”, “pulmonary disease”, “COPD”, “sinusitis”, “asthma”, “prone position”, “intubation techniques”, “chapman’s points”, and “chest wall oscillation”. The inclusion criteria were publications from 2010 to 2025 in English with full-text availability that were relevant to respiratory function, OMT, or airway management. The articles included were clinical trials and reports, experimental studies, and reviews. However, outside the 2010–2025 inclusion window, foundational texts and historically cited osteopathic resources were referenced where necessary to explain the core principles. The exclusion criteria ruled out non-peer-reviewed articles, editorials with no evidence, studies with poor methodology, studies not relevant to respiratory-directed osteopathic interventions, and animal studies, except for those that offered mechanistic relevance. In addition to the literature review, the student authors and Dr. Eller collaboratively performed and documented step-by-step demonstrations of core osteopathic techniques to ensure procedural accuracy and consistency.

## 3. Synthesis of the Literature

A total of 38 references were included in this narrative review, comprising 32 studies related to technique and mechanism explanation, 1 study supporting the narrative review methodology, and 5 background articles contextualizing the epidemiology of asthma, pneumonia, and chronic respiratory dysfunctions. These sources encompassed a range of designs, including randomized controlled trials (RCTs), observational studies, case reports, and review articles. The techniques investigated across the literature spanned craniofacial osteopathic manipulations, thoracic lymphatic approaches, emergency intubation maneuvers, and high-frequency mechanical interventions for airway support. As this narrative review follows a flexible and practical format, a PRISMA-style flowchart was not included. However, a summary of the studies is presented in Table 1 and Table 2, organized by technique, clinical focus, and key outcomes. Table 1 focuses on OMT and its physiological mechanisms supporting airway function, cranial mobility, and autonomic regulation. Table 2 highlights emergency and anesthesia-related airway techniques used for acute airway stabilization and pulmonary management. Background articles and the narrative methodology reference were excluded from Table 1 and Table 2.

### 3.1. Osteopathic Manipulative Techniques and Physiological Mechanisms

#### 3.1.1. Airway Function Improvement

The craniofacial along with cervical regions are crucial in airway function, since breathing efficiency, sinus drainage, and overall respiratory health are influenced by their structural integrity, neural pathways, and soft tissue mobility. Before describing specific techniques, understanding the third rhythmic oscillation is important because it highlights how cranial and cervical manipulations may influence deeper autonomic and lymphatic dynamics beyond structural correction alone. The third rhythmic oscillation identified in cranial studies refers to a distinct, slow intrinsic movement of the human skull, separate from respiratory and arterial pulsations, measured at an average of 6.16 cycles per minute (cpm) and aligning with the PRM proposed in cranial osteopathy [19]. While the concept of cranial rhythmic impulse (CRI) remains debated, recent literature has begun to support the presence of measurable cranial rhythms, even if these are not due to actual cranial bone movement. A 2024 systematic review by Mériaux et al. examined 28 articles and identified proposed associations between CRI and autonomic rhythms, cerebrospinal fluid dynamics, and lymphatic flow [29]. However, the review emphasized the lack of standardized protocols and statistical consistency, noting that most studies were observational and underpowered [29]. A 2025 case series by Strada et al. used visual tracking and physiologic monitoring in 20 healthy women to assess cranial dimensional changes, revealing no intrinsic cranial bone mobility but detecting subtle peripheral tissue oscillations synchronized with cardiocirculatory and respiratory rhythms, suggesting that CRI palpation may reflect broader systemic activity rather than intrinsic cranial motility [25].

**Figure 1:**
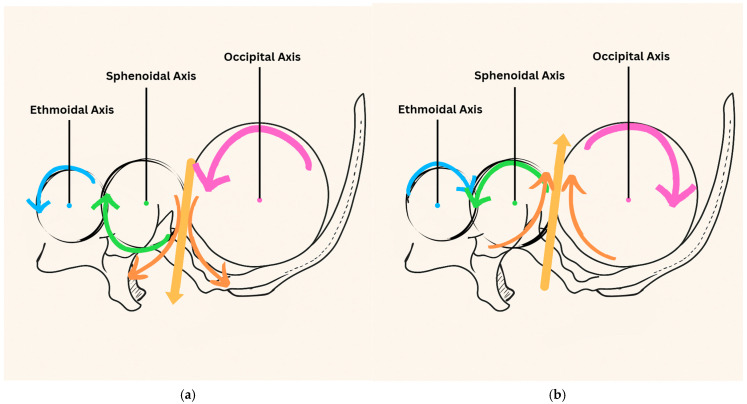
(**a**). Gear-like midline bones—extension phase. During the extension phase, the ethmoid rotates CCW. As the sphenoid is linked, it rotates CW, while the occiput also rotates CCW. This coordinated motion maintains proper tension in the falx cerebri, which is attached at the crista galli and internal occipital protuberance. (**b**). Gear-like midline bones—flexion phase. The reverse occurs in the flexion phase. The ethmoid rotates CW, the sphenoid CCW, and the occiput CW. Again, this maintains consistent falx tension.

The concept of the PRM was first introduced by William G. Sutherland in the early 20th century. He described a subtle rhythmic motion of the cranial bones, sacrum, and cerebrospinal fluid as a dynamic physiological system that could be palpated and therapeutically influenced [10]. While the PRM remains foundational in cranial osteopathy, its anatomical basis is controversial, with limited empirical support for cranial bone mobility in adults [30]. Despite ongoing debate regarding the anatomical validity of cranial bone motion, the PRM remains a core osteopathic concept. It proposes an interconnected system involving cranial bone motion, CSF, dural membrane tension, and sacral mobility to support autonomic, respiratory, and circulatory function. These interconnected dynamics—driven by the flexion and extension of midline cranial bones (sphenoid, occiput, ethmoid, and vomer), the internal and external rotation of paired cranial bones (parietals, temporals, and frontals), anchoring of the reciprocal tension membrane from the foramen magnum to S2, and a measurable cranial rhythmic impulse—form the physiological foundation for OMTs, such as the frontal lift, vomer balancing, suboccipital release, and thoracic lymphatic pumping used to enhance airway and autonomic regulation. Although the anatomical basis of cranial bone motion in fully ossified adult skulls remains controversial and is not widely accepted in mainstream literature, some osteopathic models continue to apply this framework for diagnostic and therapeutic purposes. During the cranial extension phase, the sphenoid circumducts clockwise (CW), while the ethmoid and occiput rotate counterclockwise (CCW) when viewed from the left side, demonstrating coordinated gear-like motion around their respective axes; during the flexion phase, this pattern occurs in reverse—vice versa. This biomechanical pattern is depicted in Figure 1.

In addition, the CR points located at the second intercostal spaces bilaterally correlate with the maxillary and frontal sinuses, providing another somatic–visceral diagnostic and therapeutic target for optimizing sinus drainage [8]. The palpatory findings at these points can indicate sinus congestion, and treatment of the second rib Chapman’s points can further support cranial manipulations to enhance lymphatic and immune flow through the sinonasal structures [8]. Despite the promising mechanisms, there remains a lack of consolidated literature addressing the major techniques utilized for airway function improvement with OMT. The following sections review and categorize the key craniofacial, cervical, and thoracic manipulative approaches currently described in the literature.

##### Frontal Bone Manipulation

**Figure 2:**
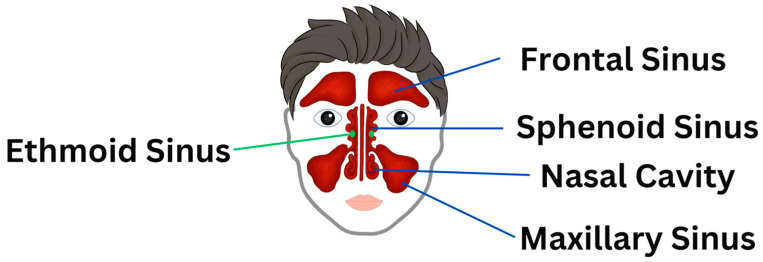
Paranasal sinuses. An anterior view of the face illustrating the anatomical positions of the frontal, ethmoid, sphenoid, and maxillary sinuses in relation to the nasal cavity.

The frontal lift OMT is commonly used in craniosacral therapy to enhance sinus drainage and support nasal cavity function, particularly benefiting the frontal sinuses, with studies suggesting it may help alleviate sinus pressure, improve nasal function, and facilitate cranial mobility [11]. Additionally, Lee-Wong et al. (2011) found that sinus OMTs, including direct pressure, “milking” techniques, and sinus drainage methods, led to a statistically significant reduction in sinus pain and congestion (*p* = 0.0012) [12]. While these techniques are often applied to maxillary sinuses, the study did not specify which sinuses were most affected [11]. Further research is needed to clarify the extent of OMT’s effects on ethmoid and sphenoid sinus drainage. Different paranasal sinuses are shown in Figure 2.

**Figure 3:**
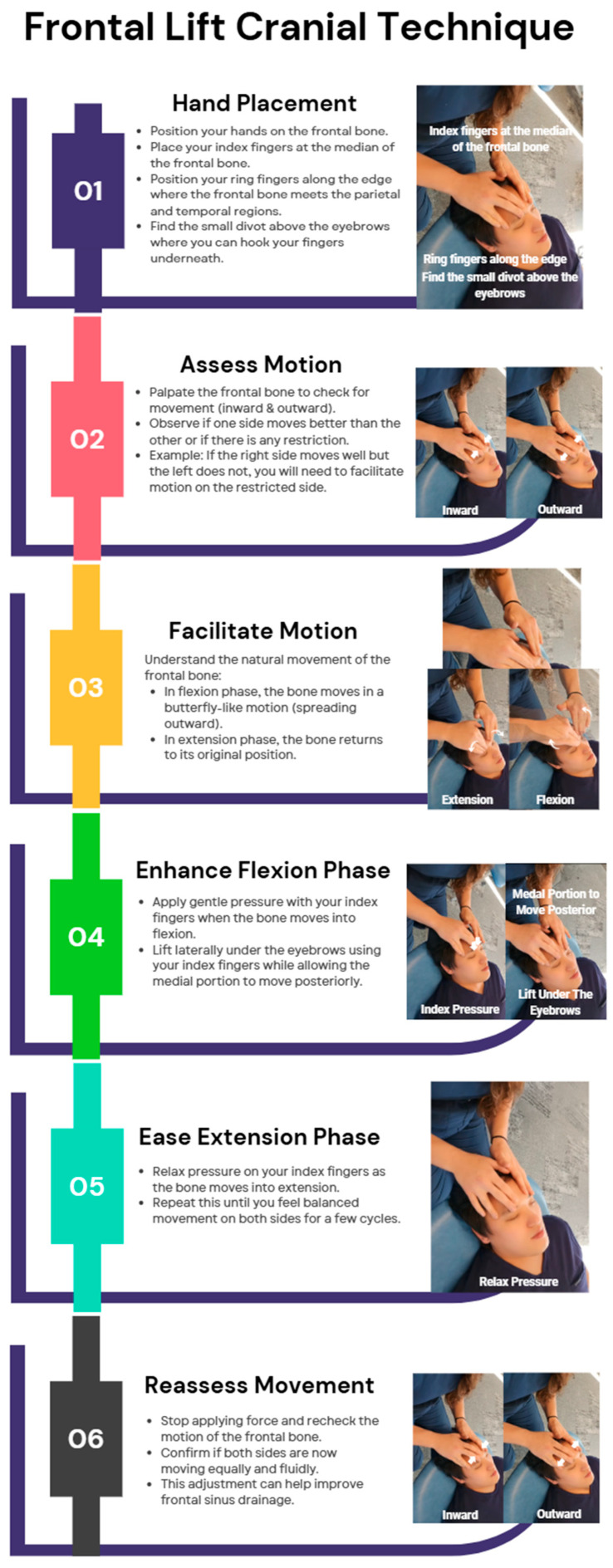
Frontal lift cranial technique. Frontal lift technique demonstrating cranial mobility assessment and sinus drainage facilitation performed by Dr. Jamie Eller, DO, on student doctor (SD) Park. This OMT aims to assess and enhance the motion of the frontal bone, promoting sinus drainage and cranial mobility. Dr. Eller follows the structured steps of hand placement, motion assessment, and movement facilitation through gentle pressure and lifting techniques. SD Park, as the recipient, is positioned comfortably while Dr. Eller skillfully applies the technique to improve cranial motion and balance.

Facial lift involves specific hand placements with the index fingers positioned on the median of the frontal bone and the ring fingers along its edge near the parietal and temporal regions, allowing for gentle palpation and motion testing to identify restrictions, followed by application of pressure during the flexion phase and relaxation during extension to enhance cranial mobility, mimicking a butterfly-like motion, ultimately facilitating sinus drainage and improving symmetry in movement. Figure 3 illustrates the step-by-step technique for performing a frontal lift OMT.

##### Vomer Manipulation

Vomer manipulation is a technique aimed at optimizing midline cranial alignment and enhancing nasal airflow, addressing dysfunctions related to nasal congestion and post-viral olfactory disturbances. A case report highlighted the positive impact of OMT, including vomer manipulation, in patients suffering from post-COVID-19 anosmia (loss of smell) and ageusia (loss of taste) [13].

##### “Nasal Walking” Technique

**Figure 4:**
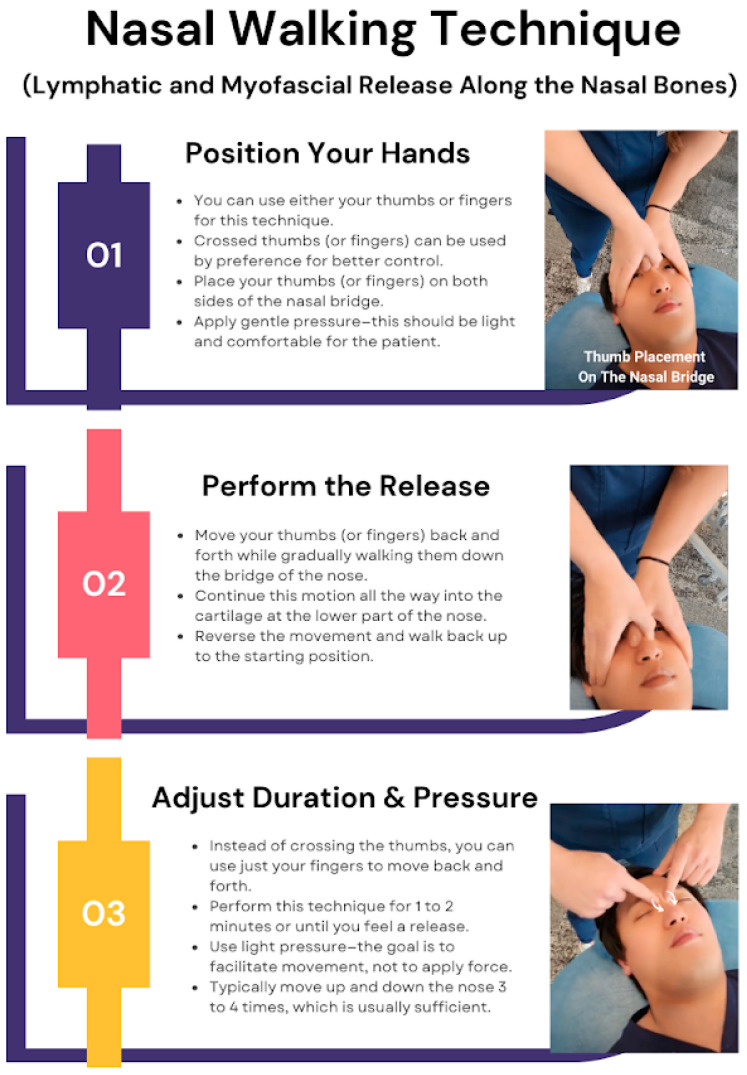
Nasal walking technique. Dr. Eller is performing the “nasal walking” technique on SD Park. This OMT focuses on lymphatic and MFR along the nasal bones to improve sinus drainage and nasal mobility. Dr. Eller carefully positions their hands, applying gentle pressure to guide SD Park’s nasal structures through a rhythmic back-and-forth motion, facilitating fascial release. With controlled adjustments in pressure and duration, Dr. Eller ensures an effective yet comfortable treatment, supporting the recipient’s nasal and cranial function. The term “nasal walking” is not officially documented in OMT literature. Instead, it is a newly created term by Dr. Eller and SD Park used to describe this specific combined cranial, lymphatic, and MFR technique along the nasal bones. The name originates from the walking-like motion of the physician’s thumbs or fingers as they rhythmically move along the nasal bridge and cartilage.

The first technique demonstrated is a combination of lymphatic and myofascial release (MFR) along the nasal bones to facilitate side strain relief. To begin, the practitioner can use their thumbs or any fingers that feel most comfortable. A preferred method involves crossing the thumbs for better control and maintaining a more rhythmic motion. The technique starts by placing the thumbs on both sides of the nasal bridge and applying gentle pressure while moving them back and forth, gradually walking along the bridge of the nose. This motion is continued down into the cartilage before reversing back up. The technique should be performed for about one to two minutes or until a release is felt, ensuring that the pressure remains light and comfortable for the patient. Crossing the thumbs helps maintain a more rhythmic motion and makes the technique easier to control. Typically, moving up and down the nose three to four times is sufficient for a beneficial response. This technique, referred to here as “nasal walking”, is demonstrated in Figure 4.

##### Vomer Balancing Technique

**Figure 5:**
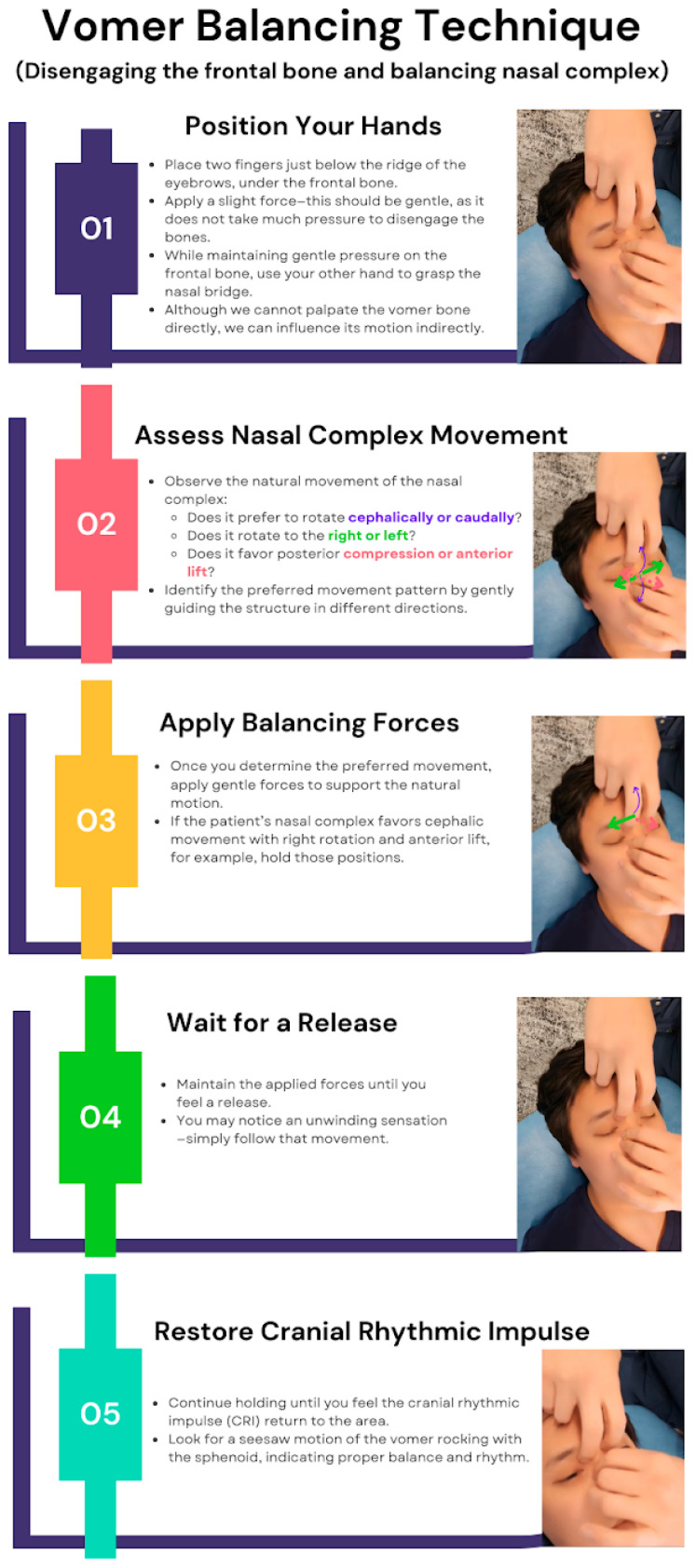
Vomer balancing technique. Dr. Eller is performing the “vomer balancing” technique on SD Park, showing hand positioning, assessment of nasal complex motion, application of balancing forces, release phase, and restoration of cranial rhythmic impulse.

Another technique, vomer balancing, is a recognized cranial OMT that can be used to further facilitate sinus drainage. This approach begins by disengaging the frontal bone with gentle pressure applied just below the eyebrow ridge. A slight force is used—not overly strong—to subtly influence the nasal complex. The physician then stabilizes the nasal bridge to indirectly affect the vomer’s motion. Since the vomer itself cannot be directly palpated, its movement is assessed by observing whether the nasal complex favors a superior, inferior, rightward, or leftward rotation, as well as whether it has a tendency toward posterior compression or anterior lift. Once the preferred motion pattern is identified, the practitioner applies a combination of these forces and holds them until a release is felt. Sometimes, an unwinding sensation is noticeable, at which point the practitioner continues to follow the motion until the CRI returns, defined as a subtle, palpable rhythm (typically 6–12 cycles per minute) that reflects the inherent motion of CSF, meningeal tension, and cranial structures. The objective is to restore optimal cranial rhythm and function by re-establishing the vomer’s natural seesaw motion as it synchronizes with the sphenoid bone. The “vomer balancing” technique is shown in Figure 5.

A case study demonstrated significant symptom relief in a chronic rhinosinusitis (CRS) patient following OMT, with improvements in nasal patency, facial pressure, and overall QoL [14]. When applied appropriately, OMT can alleviate symptoms such as CRS by enhancing sinus drainage, improving nasal airflow, and addressing cranial restrictions [14].

##### Zygomatic Bone Manipulation

**Figure 6:**
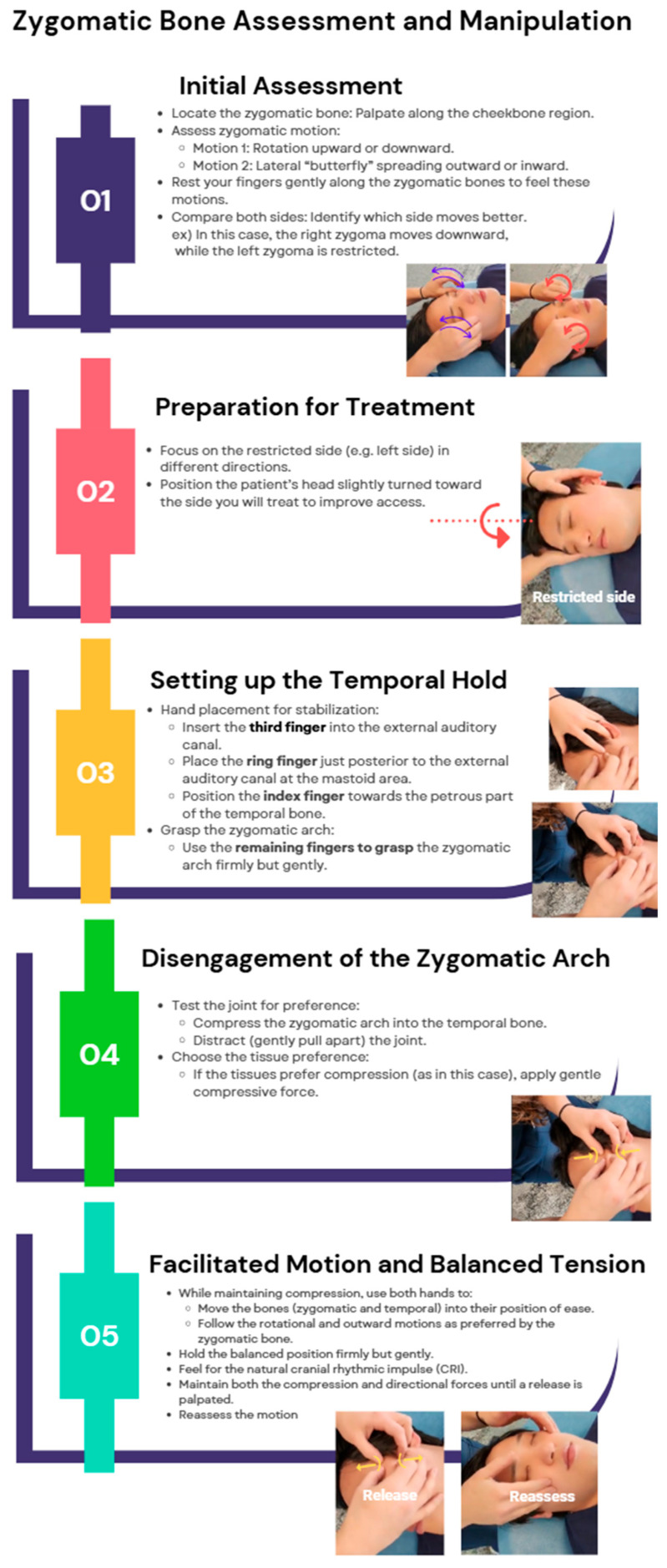
Zygomatic bone technique. Dr. Eller is performing the zygomatic bone assessment and treatment on SD Park, showing hand positioning, assessment of motion, application of balancing forces, release phase, and restoration of cranial rhythmic impulse.

By improving midface structural support, the zygomatic bone manipulation technique may help reduce nasal resistance. The primary management of maxillofacial trauma involves addressing challenges in securing a patent airway [15]. OMT has been shown to enhance upper airway stability, improve airway function, and support respiratory health, highlighting its potential role in both trauma management and broader airway stabilization strategies [16]. Although there is no literature directly supporting the role of zygomatic manipulation in managing airway complications, assessing and adjusting zygomatic motion can help restore balance in cranial mechanics, as the zygoma exhibits both rotational and butterfly-like outward movement, influencing the surrounding facial structures and their function. Proper disengagement and mobilization of the zygomatic arch can improve overall CRI, contributing to better structural alignment and functional airway support. The zygomatic bone manipulation technique is shown in Figure 6.

##### Maxillary Bone Manipulation

**Figure 7:**
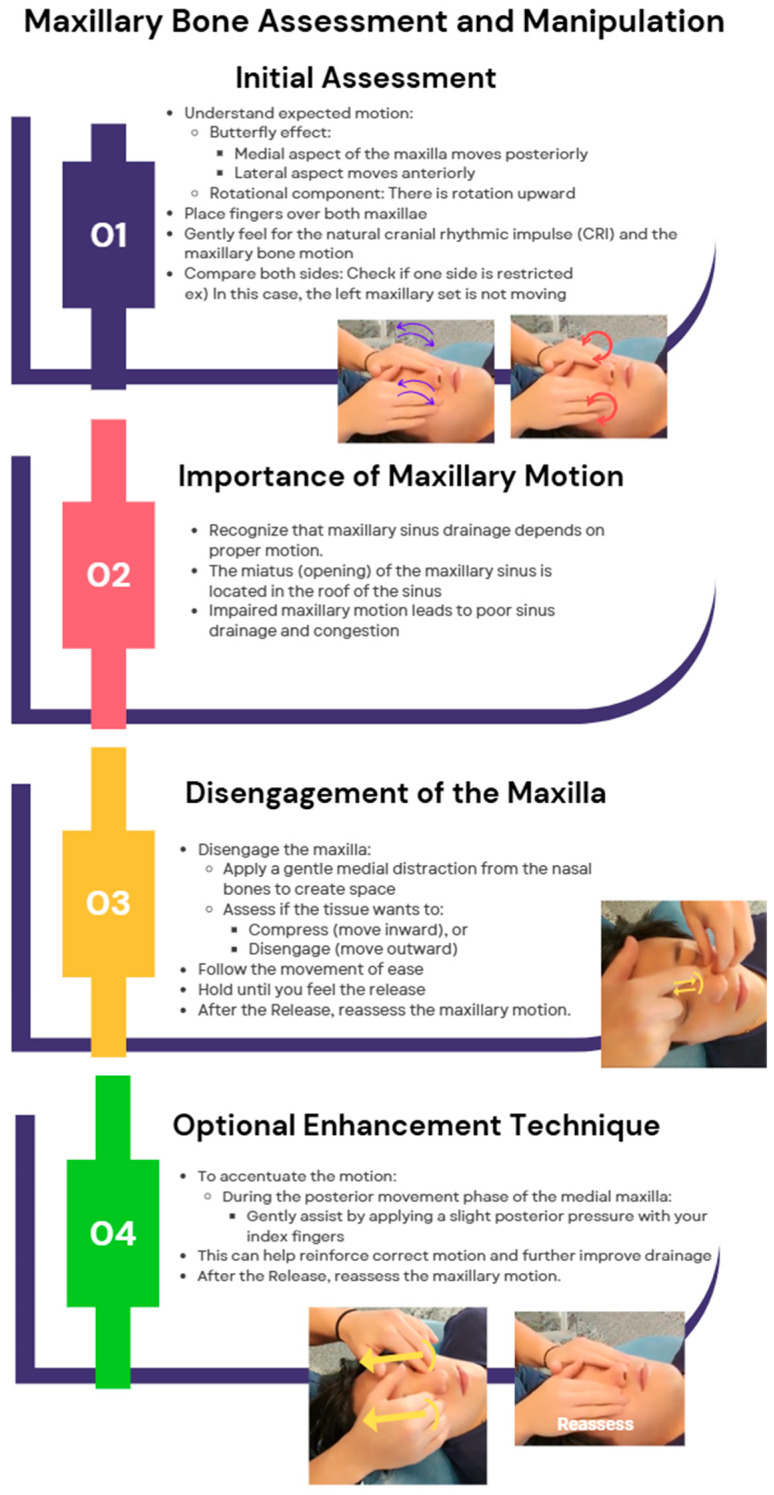
Maxillary bone technique. Dr. Eller is performing the maxillary bone assessment and treatment on SD Park, showing hand positioning, assessment of motion, application of balancing forces, release phase, and restoration of cranial rhythmic impulse.

To assess and improve maxillary motion using the concept of balanced membranous tension, begin by understanding its movement, which follows a butterfly effect, where the medial aspect moves posteriorly, the lateral aspect moves anteriorly, and there is an upward rotational component. Initiate treatment by placing your hands on the maxilla to evaluate its motion in comparison to the frontalis and zygoma, identifying any restrictions, as maxillary mobility is crucial for sinus drainage due to the miatus being located at the roof of the sinus, where poor movement can lead to ineffective drainage and congestion. To release restrictions, establish a proper hand placement on the maxilla and gently disengage it from the nasal bones by applying medial distraction, creating space to determine whether the maxilla wants to compress or disengage. If it tends to compress, follow this motion and then guide it toward ease until a floating balance point is achieved. Hold this position until a release occurs, then continue following the motion for a few cycles to ensure proper mobility. After reassessment, if additional enhancement is needed, gently accentuate the motion using your index fingers by encouraging the medial aspect of the maxilla to move posteriorly when appropriate. This structured approach restores maxillary mobility, promoting better sinus drainage and overall cranial balance. The maxillary bone manipulation technique is shown in Figure 7.

##### C2 and Cervical Manipulations

Cervical manipulation techniques involve inducing translation (left and right), sidebending, and rotation to assess the movement of ease of the fascia, aiming to enhance vagal tone and reduce airway resistance by correcting cervical somatic dysfunctions, as this region contains the vagus nerve and major lymphatic drainage pathways [17]. Particular attention should be paid to the occipitoatlantal (OA) and atlantoaxial (AA) junctions—addressed through suboccipital inhibition, OA decompression (OA-D), and balanced ligamentous tension—which demonstrate short-term enhancement of parasympathetic tone through increased HRV [18]. The structural realignment with fascial balance may theoretically support longer-term normalization of vagal regulation. However, longitudinal studies are needed to confirm this effect.

**Figure 8:**
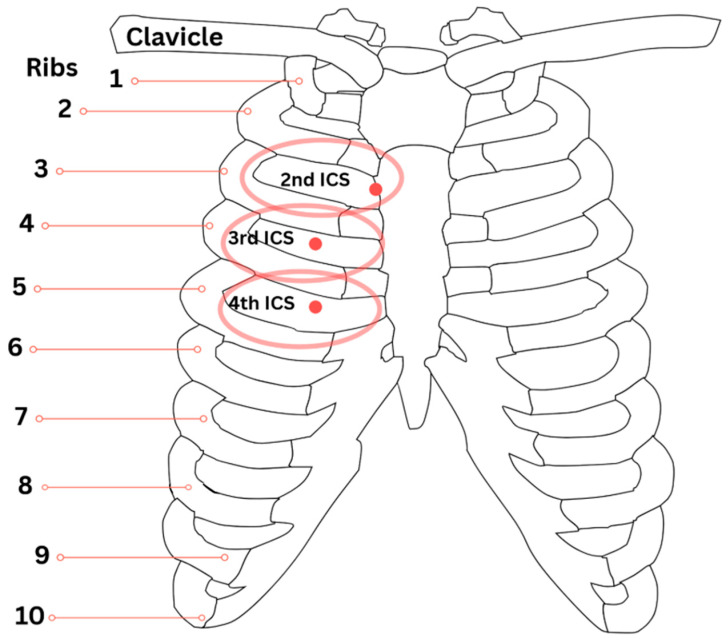
Anatomical locations of Chapman’s reflex points for sinus and pulmonary function. This illustration highlights the anterior Chapman’s points associated with sinus and lung drainage. The second ICS points, located just lateral to the sternum at the sternocostal junction, correspond to sinus congestion. The third ICS points reflect dysfunction of the upper lungs, while the fourth ICS points correspond to the lower lungs. Each point is positioned slightly lateral to the midline along the costosternal junction, providing a reproducible somatic–visceral diagnostic landmark for respiratory conditions.

Although the cervical spine primarily influences autonomic regulation through the vagus nerve, somatic dysfunctions at cervical levels may contribute to thoracic and pulmonary impairments through dural attachments, lymphatic congestion, and altered neural signaling [8,17]. Lymphatic congestion in particular may manifest as palpable CR points, serving as both diagnostic and therapeutic landmarks for respiratory dysfunction. Relevant CRs include the upper lung points located at the medial third intercostal spaces, lower lung points at the medial fourth intercostal spaces, and posterior lung points at the T3 and T4 transverse processes, all of which highlight the somatic–visceral link between cervical integrity and pulmonary health [8]. These points are anatomically consistent and reproducible between individuals. Figure 8 illustrates the typical anatomical locations of the second, third, and fourth ICS Chapman’s points associated with sinus and lung function.

#### 3.1.2. Osteopathic Manipulative Treatments for Pneumonia—Thoracic Soft Tissue Techniques

The thoracic cavity and pulmonary structures are integral to breathing techniques and may contribute to patients’ symptoms involving cough, dyspnea, tachypnea, sputum production, chest pain, and pneumonia [20]. This review introduces a couple of OMTs appropriate for supporting pneumonia management. However, it is strongly recommended to address and correct all somatic dysfunctions before initiating these targeted treatments.

##### Doming the Diaphragm to Facilitate Diaphragmatic Motion and Rib Raising

In this technique, the patient lies supine while the physician palpates the base of the patient’s ribs, assessing bilateral thoracic cage motion during inhalation and exhalation. Instruct the patient to take a deep breath and ensure that both thumbs are about one inch inferolateral to the xiphoid process, resting on the anterolateral costal margin below rib seven. The remaining digits should rest along the inferolateral border of ribs eight through ten. As the patient exhales, the physician follows the diaphragm by pressing both thumbs posteriorly toward the table and cephalad. This pressure should be maintained as the patient takes the next inhalation. During the subsequent exhalation, the physician should press the thumbs further cephalad within reasonable limits, taking care to avoid patient discomfort. This cycle should be repeated for three to five respiratory cycles, after which diaphragmatic motion should be reassessed by repositioning the hands over the ribs to monitor improvement in excursion. The expected result is for the diaphragm to easily dome at the end of exhalation. Reassessing diaphragmatic motion can be achieved by repositioning the hands over the ribs to monitor the improvement in excursion. Furthermore, “the diaphragm assists lymphatic flow by exerting a pump-like propulsion effect on fluid within vessels” [20]. Rib raising augments lymphatic flow by improving respiratory excursion and perfusion in the thoracic walls. Rib-raising groups were found to have decreased levels of α-amylase, an established biomarker of sympathetic activity, compared to a light-touch control group [20].

##### Thoracic Lymphatic Pump with Respiratory Assist

**Figure 9:**
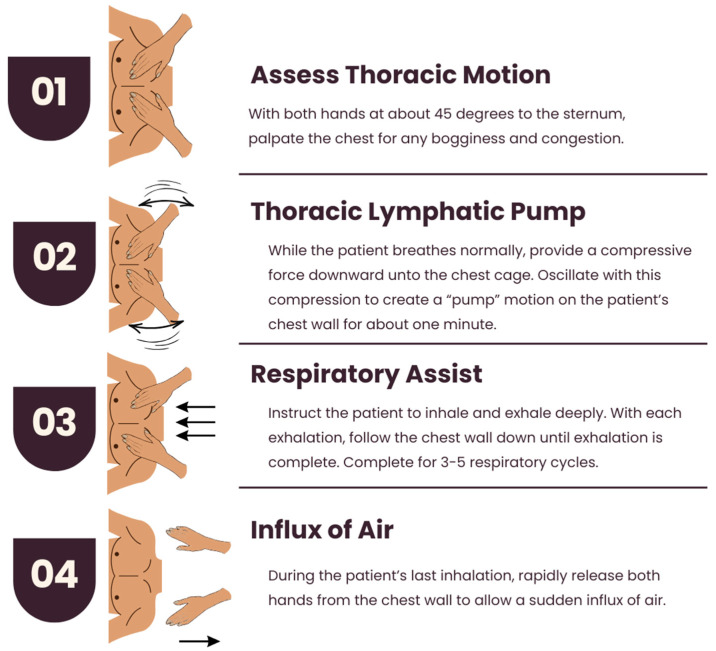
Thoracic lymphatic pump with respiratory assist. The thoracic lymphatic pump with respiratory assist involves assessing thoracic motion for bogginess, applying oscillating compressive force to the chest wall, coordinating deep breathing with compression over three to five respiratory cycles, and rapidly releasing both hands during the final inhalation to promote lymphatic drainage.

The patient should lie supine, and the table should be adjusted to be at arm’s length by the physician at the head of the table. The thoracic chest motion may reveal congestion and bogginess. Both hands should be placed over the pectoral region, with the palms just distal to the clavicles and the thumbs at about 45 degrees to the sternum. While the patient breathes normally, provide a compressive force downward on the chest cage. With this force, oscillate the degree of compression to produce a pump motion on the patient’s chest wall for approximately one minute or until proper lymphatic flow is achieved. Next, you may initiate respiratory assist by instructing the patient to inhale and exhale deeply. With each exhalation, follow the chest wall down until exhalation is complete. Maintain both hands on the chest wall, and in the next exhalation phase, press down on the chest wall again. This should be repeated for three to five cycles, each time compressing the patient’s chest wall inferiorly. During the patient’s last inhalation, rapidly release both hands from the chest wall to allow for a sudden influx of air. The thoracic motion should be reassessed after completing the treatment [18]. Figure 9 illustrates the “thoracic lymphatic pump with respiratory assist” technique.

#### 3.1.3. Osteopathic Manipulative Treatments for Asthma

**Figure 10:**
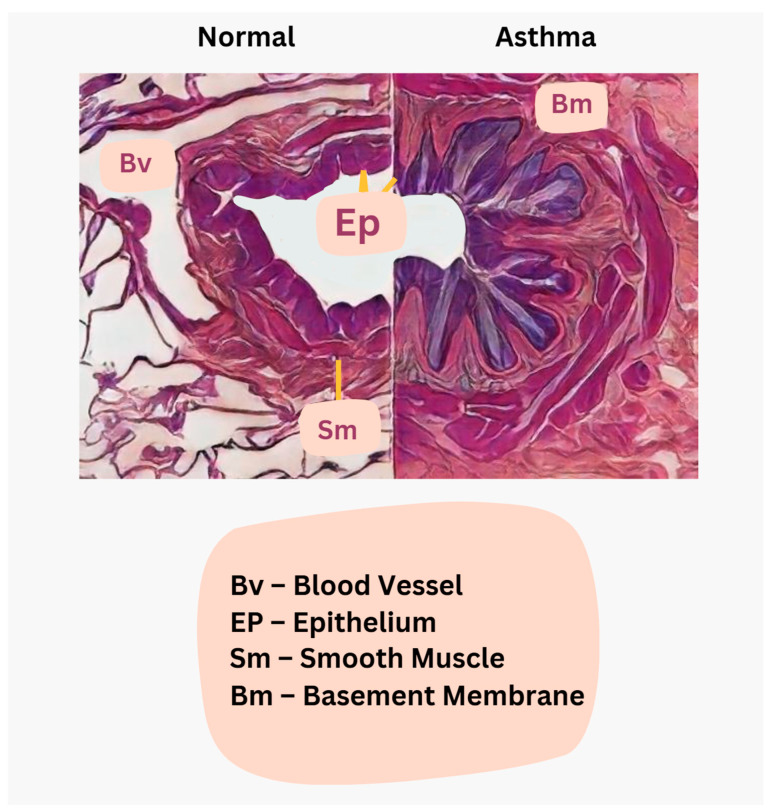
Histological illustration for comparison: Normal vs. asthma airways. In a normal airway, the lumen remains wide for smooth airflow; the epithelium (Ep) is intact and well organized; the smooth muscle (Sm) layer is thin to maintain normal airway tone; the blood vessels (Bv) are evenly distributed without inflammation; and mucus production is minimal to prevent obstruction. However, in asthmatic conditions, the airway displays sm hypertrophy, a narrower lumen, and a thickened basement membrane (Bm) due to fibrosis, restricting the airway and contributing to bronchospasm and airway constriction.

In asthma, IgE-mediated inflammatory infiltration with eosinophils and Charcot–Leyden crystals contributes to mucus plugging, while goblet cell hyperplasia leads to excessive mucus production [24]. Epithelial damage results in squamous metaplasia, and submucosal gland enlargement further increases mucus secretion, all of which, combined with autonomic dysregulation, drive bronchoconstriction and airway hyperreactivity, manifesting as the hallmark symptoms of asthma [23,24]. Emerging insights have also revealed that epithelial–mesenchymal crosstalk and persistent inflammatory signaling involving Th2 and Th17 cells, innate lymphoid cells (ILC2), and associated cytokines like IL-4, IL-5, and IL-13 contribute to both inflammation and airway remodeling processes, rather than inflammation alone being the sole driver [23,24]. The histological comparison between normal and asthma airways is shown in Figure 10.

Schend et al. (2020) presented a modular framework for osteopathic asthma care that integrates rib raising to improve thoracic cage mobility and sympathetic regulation, thoracic lymphatic pump techniques to enhance immune drainage and mucociliary clearance, and cranial techniques—including frontal lift and occipitoatlantal decompression—to promote parasympathetic tone and autonomic balance [21]. The review emphasized both historical cases of osteopathic success in asthma and contemporary relevance through video-based instruction for clinical replication [21]. Meanwhile, Jones et al. (2021) conducted a RCT in pediatric asthma patients, demonstrating non-significant improvements in pulmonary function following rib-raising and suboccipital release OMT. The OMT group showed greater mean increases across several spirometry measures (forced vital capacity (FVC), forced expiratory volume in 1 s (FEV_1_), FVC/FEV_1_ ratio, and forced expiratory flow at 25–75% of pulmonary volume (FEF 25–75%)) compared to standard care, but statistical significance was not reached (exact *p*-values not provided) [22].

While no conclusive outcome data currently support a significant impact of OMT on asthma, related studies in other respiratory conditions—such as CRS and chronic obstructive pulmonary disease (COPD)—and in healthy individuals have demonstrated measurable physiologic benefits. Rowane et al. (2024) found that a targeted cranial and thoracic OMT protocol significantly reduced nasal congestion (*p* = 0.001), postnasal drainage (*p* = 0.002), and sinus pressure (*p* = 0.0004) in patients with CRS [26]. Stępnik et al. (2020) reported a statistically significant increase in peak expiratory flow (*p* < 0.001) following a single OMT session in healthy adults, although no changes were observed in FEV1 or FVC [27]. In patients with severe COPD, Zanotti et al. (2011) showed that adding OMT to pulmonary rehabilitation significantly improved exercise capacity (Δ = +72.5 m, *p* = 0.01) and reduced residual lung volume (Δ = –0.44 L, *p* = 0.001) compared to rehabilitation alone [28]. These findings suggest a potential supportive role for OMT in enhancing respiratory function across multiple contexts, although asthma-specific data remain limited.

### 3.2. Emergency-Anesthesia Maneuvers Related to Airway Management, Pneumonia, and Asthma

**Figure 11:**
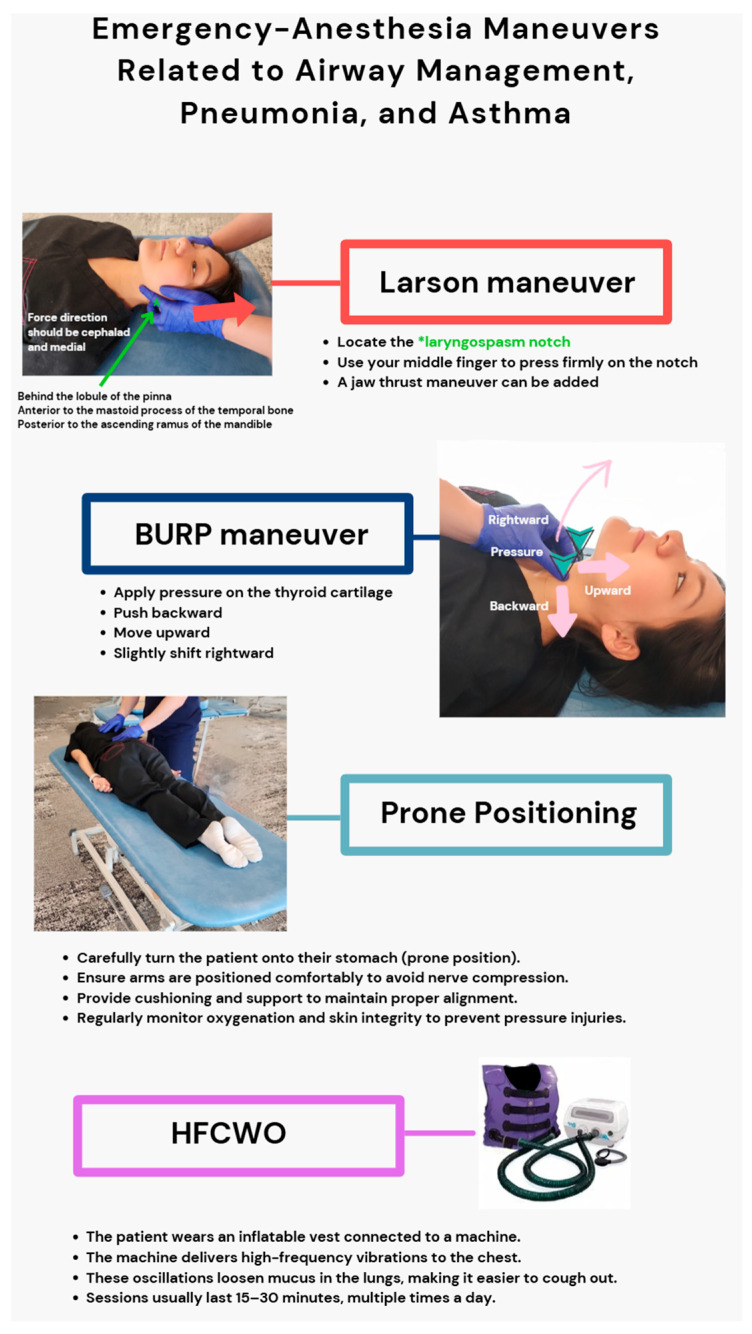
Emergency airway management technique. SD Park was performing maneuvers on SD Benitez to demonstrate the common manual or device-mediated respiratory techniques, including the Larson maneuver, BURP maneuver, prone positioning, and high-frequency chest wall oscillation (HFCWO), highlighting their applications in optimizing ventilation.

To facilitate adequate oxygenation and ventilation in emergency airway situations, management techniques such as the Larson maneuver, which involves applying bilateral pressure over the “laryngospasm notch” to overcome laryngospasm, and the backward, upward, rightward pressure (BURP) maneuver, which facilitates augmented glottic visibility during intubation, are essential for effective airway protection and intubation facilitation [31,33]. The prone position enhances oxygenation in acute respiratory distress syndrome (ARDS) by allowing more even distribution of ventilation, reducing dorsal lung compression, avoiding ventilation–perfusion mismatch, and decreasing airway resistance in pneumonia and asthma [34,35]. Interestingly, an alternative approach involving manual manipulation or postural modification through a mechanical device—high-frequency chest wall oscillation (HFCWO)—enhances mucoclearance and lowers airway resistance in asthma disease and improves pulmonary mechanics in pneumonia-induced respiratory failure by facilitating secretion mobilization [36,37]. The general instructions and visualization of each technique are illustrated in Figure 11.

These airway management techniques focus on immediate mechanical and physiological interventions for optimizing ventilation, while OMTs sustain holistic treatment to enhance structural alignment and autonomic balance over time. Among these, prone positioning has demonstrated statistically significant mortality reduction in ARDS (Yan et al., 2025; HR 0.53; 95% CI, 0.32–0.85; *p* = 0.033) [38]. HFCWO has shown immediate improvements in tidal volume, oxygenation, and peak airway pressure in mechanically ventilated patients with pneumonia (*p* < 0.05 for tidal volume, oxygenation) and has contributed to symptom resolution in refractory asthma [36,37]. In contrast, techniques such as BURP and Larson’s maneuver are widely used but are primarily supported by case-based and observational evidence, lacking statistically validated outcomes [31,32]. OMT remains an emerging adjunct with preliminary physiologic support but lacks comparable high-level clinical trial data. These modalities differ in urgency, mechanism, and evidentiary foundation and should be interpreted accordingly in clinical contexts.

## 4. Discussion

The findings of this review must be interpreted in the context of several limitations. First, the number of included studies (n = 38) is relatively small, and study designs were heterogeneous, many being case reports or observational studies rather than randomized controlled trials. Second, this review used only PubMed and Google Scholar as data sources, which may have excluded relevant literature indexed in other major databases, such as Embase, CINAHL, or Cochrane Library. This narrow search scope limits the comprehensiveness and may introduce selection bias. Furthermore, variation in OMT practitioner techniques and lack of standardized protocols across studies reduce reproducibility and make the generalization of findings challenging. Future reviews and trials should prioritize standardized protocols, broader database use, and multicenter trials to confirm the efficacy of these interventions.

The mentioned cranial techniques, such as frontal bone, vomer, zygomatic, and maxillary manipulations, aim to normalize elevated parasympathetic tone. However, in the OMT for asthma, the focus is on indirectly enhancing thoracic compliance and activating the sympathetic chain to optimize autonomic regulation and improve airway function. Chapman’s points located bilaterally in the third and fourth intercostal spaces should be evaluated and treated, as they are associated with pulmonary reflexes and can reflect bronchial and upper lung dysfunction [5]. Correction of somatic dysfunctions at the OA and AA junctions is crucial due to their dural connections and significant influence on vagal tone [17,18]. Further, addressing dysfunctions in the C2–C4 region can improve diaphragmatic function via the phrenic nerve. While cranial OMTs, such as suboccipital decompression and occipito-mastoid suture manipulation, have demonstrated short-term enhancement of parasympathetic activity, evidenced by increased heart rate variability, their potential to promote longer-term normalization of vagal tone may be mediated through structural realignment, reduction in fascial tension, and enhanced lymphatic drainage.

**Figure 12:**
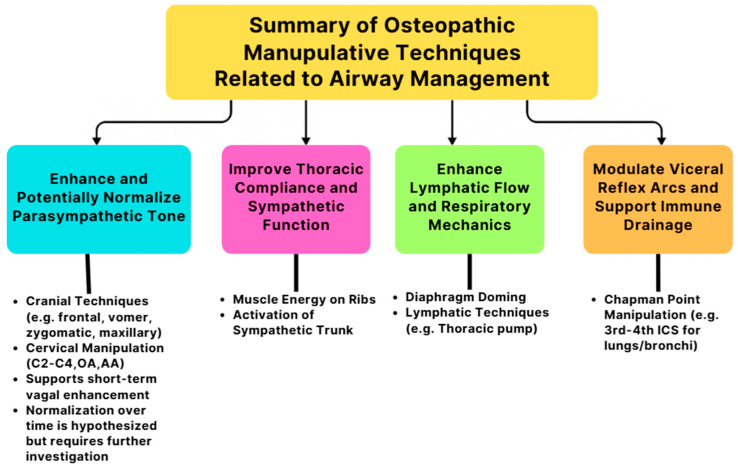
The four key domains of OMTs that support autonomic and respiratory balance by stabilizing parasympathetic tone through cranial and cervical manipulations; improving thoracic compliance and sympathetic function via rib-focused techniques; enhancing lymphatic flow and respiratory mechanics through diaphragm doming and thoracic pump; and modulating visceral reflex arcs and immune drainage with Chapman’s point manipulation.

Rib dysfunctions can be managed using ME techniques to restore thoracic compliance and support autonomic ganglia chains along the sympathetic trunk, promoting bronchodilation. Specific somatic dysfunctions in cervical or throacic, such as left rotation of C3 and any issues isolated to T3, should also be corrected to ensure balanced neural input and mechanical integrity for optimal respiratory mechanics. These interventions together support enhanced lymphatic flow, decreased airway resistance, and improved vagal modulation, especially in conditions like asthma and pneumonia. Figure 12 summarizes how OMTs support autonomic and respiratory balance by targeting four key domains.

Airway hypersensitivity seen in asthma involves mast cells (tissue residents) and basophils (circulation) both releasing histamine and inflammatory mediators that contribute to inflammation and symptom exacerbation, while IgE, produced in the germinal center through somatic hypermutation of IgM, plays a key role in the immune response. This raises a question: would lymphatic techniques help attenuate this hypersensitivity by enhancing immune cell drainage, reducing inflammatory mediator buildup, or modulate to activate the somatic hypermutation and class-switching process within lymphoid tissues? A more in-depth investigation is needed to explore these mechanisms, and the study of CRs may offer valuable insight into how somatic–visceral interactions influence immune modulation and airway hypersensitivity. Findings related to CRs further underscore the somatic–visceral connection and justify the integration of osteopathic interventions in respiratory care. Emerging evidence suggests that craniofacial and cervical structures function as an integrated physiological network influencing autonomic and respiratory dynamics, while temporal bone motion has been proposed to affect Eustachian and intracranial pressure regulation [7,11,30]. However, further investigation is needed.

Despite the growing interest, there remains a notable gap in the literature regarding direct studies of zygoma OMT, maxillary bone manipulation, and standardized investigation of Chapman’s reflex points. The current evidence is largely extrapolated from broader cranial or visceral techniques rather than specific, isolated interventions. Cranial base restrictions, particularly near the jugular foramen, may compromise cranial nerves IX and X, contributing to upper airway dysfunctions, such as dysphagia, impaired cough reflex, and altered airway tone. Further research is essential to clarify the precise role of these manipulations and OMT in respiratory and autonomic function.

This review is limited by the heterogeneous nature of the available literature, the absence of large-scale RCTs on craniofacial and cervical techniques, the reliance on case reports and observational studies, and practitioner variability in OMT delivery, all of which constrain reproducibility and generalizability. Despite these limitations, this review provides an organized summary of maneuvers that improve airway management through OMT and offers a comprehensive overview of current evidence, emphasizing the need for future clinical trials with standardized outcome measures.

## 5. Conclusions

While high-level clinical efficacy data remain limited, this narrative review synthesizes the emerging physiologic, anatomical, and mechanistic evidence, suggesting that OMTs, including craniofacial, cervical, thoracic, and lymphatic interventions, may play a valuable role in respiratory care by enhancing airway patency, autonomic regulation, and immune drainage. Notable findings—such as reduced nasal resistance (Rowane et al., 2024), improved expiratory flow (Stępnik et al., 2020), and enhanced exercise capacity in COPD (Zanotti et al., 2011)—demonstrate measurable physiologic benefits of OMT [26,27,28]. Additional studies (Mériaux et al., 2024; Strada et al., 2025) show that OMT can modulate autonomic tone and lymphatic flow, supporting its role as a complementary approach in respiratory care [25,29]. Through vagal stimulation, improved lymphatic return, and structural airway stabilization, these interventions offer a compelling, multimodal strategy for respiratory care. Mapping CRs serves as a valuable diagnostic tool for identifying somatic dysfunctions that contribute to airway compromise. Craniofacial and cervical manipulations, thoracic OMT, and emergency airway maneuvers together represent a comprehensive approach that offers both immediate and long-term enhancement of structural, neurological, and immune aspects of airway function. Although the current evidence supports the role of OMT in respiratory care, significant gaps—particularly in zygomatic and maxillary bone manipulation and Chapman’s point validation—emphasize the need for standardized protocols and further clinical trials to fully integrate these techniques into broader care models for asthma, pneumonia, and airway dysfunction.

## Figures and Tables

**Table 1:** OMT and physiological mechanisms.

Author (Year)	Technique Studied	Condition/Focus	Study Type	Key Outcomes
Bath et al. (2023) [8]	Chapman’s Reflex Points	Sinus drainage, respiratory lymphatic support	Review	Described neuro-lymphatic reflexes; highlighted 2nd rib points for sinus drainage, 3rd and 4th ICS points for sinus and pulmonary drainage
Lintonbon (2019) [11]	Frontal lift OMT	Chronic Sinusitis	Case Study	Improved sinus drainage and cranial mobility
Lee-Wong et al. (2011) [12]	Sinus OMTs	Chronic Sinusitis	Observational	Statistically significant relief (*p* = 0.0012)
Mehra et al. (2024) [13]	OMT including vomer manipulation	Post-COVID anosmia & ageusia	Case Report	Sense of smell and taste improvement
Baisakhiya (2020) [14]	Vomer and Sinus manipulation	Chronic Rhinosinusitis (CRS)	Case Report	Improved nasal airflow, sinus drainage
Saini et al. (2021) [15]	Airway management in maxillofacial trauma	Maxillofacial trauma, airway stabilization	Review/Perspective	Facilitated airway management, facial structural alignment; no direct OMT technique evaluated
King (2018) [16]	OMT for upper airway stabilization	General airway function	Review/Perspective	Noted improvement in airway stability
Giles et al. (2013) [17]	Suboccipital decompression	Vagal tone, Heart Rate Variability (HRV)	RCT	Increased HRV, improved autonomic balance
Dalgleish et al. (2021) [18]	Occipitoatlantal decompression (OA-D)	Vagal tone modulation, cardiac autonomic control	RCT	Prolonged AV conduction and increased HRV, suggesting enhanced vagal tone after OA-D intervention
Rasmussen & Meulengracht (2021) [19]	Cranial rhythmic motion	Physiological mechanism	Biomechanical Study	Identified distinct cranial rhythm (~6.16 cpm), supports cranial osteopathy basis
Yao et al. (2014) [20]	Thoracic lymphatic pump, rib raising	Pneumonia	Experimental	Increased lymphatic flow, reduced congestion
Anwar et al. (2022) [7]	Breathing reeducation	Chronic neck pain & pulmonary outcomes	RCT	Improved cervical and pulmonary function
Chu et al. (2023) [6]	Cervical radiculopathy	Angina, dyspnea	Case Report	Linked cervical dysfunction to cardiopulmonary symptoms
Schend et al. (2020) [21]	Modular OMT techniques	Asthma management	Narrative Review	Outlined OMT principles, historical context, and practical techniques for asthma patients
Jones et al. (2021) [22]	Rib raising and suboccipital release	Pediatric asthma	RCT	Improved pulmonary function testing (PFT) values; however, changes not statistically significant
Lambrecht & Hammad (2015) [23]	Immunopathology of airway hypersensitivity	Asthma inflammation mechanisms	Review	Described Th2 and Th17 pathways, ILC2 cells, eosinophilic vs neutrophilic asthma heterogeneity
Hough et al. (2020) [24]	Epithelial-mesenchymal interactions	Asthmatic airway remodeling	Review	Described airway remodeling processes, matrix deposition, airway smooth muscle proliferation, inflammaging concept
Strada et al. (2025) [25]	Cranial dimensional motion	Cranial Rhythmic Impulse – systemic activity	Case Series	Subtle peripheral oscillations correlated with cardiopulmonary rhythms; CRI may reflect systemic physiological activity rather than cranial bone movement.
Rowane et al. (2024) [26]	Cranial and thoracic OMT	Chronic rhinosinusitis (CRS)	RCT	Statistically significant reduction in nasal congestion (*p* = 0.001), postnasal drainage (*p* = 0.002), and sinus pressure (*p* = 0.0004).
Stępnik et al. (2020) [27]	OMT techniques	Healthy subjects—pulmonary function	RCT	Increased peak expiratory flow (*p* < 0.001); no significant changes in FEV1 or FVC.
Zanotti et al. (2011) [28]	OMT adjunct to pulmonary rehab	Severe COPD	Pilot Study	Improved exercise capacity (+72.5 m, *p* = 0.01); decreased residual lung volume (–0.44 L, *p* = 0.001) vs. rehab alone.
Mériaux et al. (2024) [29]	Cranial rhythmic impulse (CRI) & PRM	PRM physiology and autonomic regulation	Systematic Review	Identified link between CRI, autonomic activity, and CSF flow; emphasized methodological heterogeneity across studies.
Sutherland (1939) [10]	Primary Respiratory Mechanism (PRM)	Osteopathic cranial model foundation	Foundational Educational Text	Introduced concept of cranial rhythmic motion and CSF dynamics forming a unified physiological system.
Rogers & Witt (1997) [30]	Cranial bone motion	Anatomical validity of cranial motion	Literature Review	Identified limited evidence for adult cranial bone mobility; called for more outcomes-based research.

**Table 2:** Emergency and anesthesia-related airway techniques.

Author (Year)	Technique Studied	Condition/Focus	Study Type	Key Outcomes
Hill et al. (2019) [31]	Larson’s maneuver	Laryngospasm during intubation	Case Technique Report	Improved intubation during laryngospasm
Yu et al. (2020) [32]	BURP maneuver	Difficult laryngoscopy/intubation	Observational Study	Reduced difficult laryngoscopy from 21.1% to 6.1% (*p* < 0.005)
Oh et al. (2021) [33]	BURP maneuver	Difficult intubation	Retrospective	Improved glottic visualization
Gattinoni et al. (2023) [34]	Prone positioning	* ARDS	Review	Improved oxygenation, reduced ventilation-perfusion mismatch
Harris et al. (2009) [35]	Prone positioning	Bronchoconstriction/Asthma	Experimental	Reduced ventilation defects, better lung expansion
Bose et al. (2013) [36]	* HFCWO	Refractory asthma	Case Study	Helped control symptoms not responsive to drugs
Chuang et al. (2017) [37]	* HFCWO	Pneumonia-induced respiratory failure	RCT	Improved secretion clearance and pulmonary mechanics
Yan et al. (2025) [38]	Prone positioning	* ARDS	Retrospective Cohort	Mortality benefit (HR 0.53; 95% CI 0.32–0.85; *p* = 0.033)

* A list of abbreviations is provided at the end.

## Data Availability

No new data were created or analyzed in this study. Data sharing is not applicable to this article.

## Abbreviations

The following abbreviations are used in this manuscript:

AA	Atlantoaxial (Joint)
ARDS	Acute Respiratory Distress Syndrome
Bm	Basement Membrane
Bv	Blood Vessel
BURP	Backward, Upward, Rightward Pressure
CI	Confidence Interval
COPD	Chronic Obstructive Pulmonary Disease
cpm	Cycles Per Minute
CRI	Cranial Rhythmic Impulse
CR(s)	Chapman’s Reflex(es)
CRS	Chronic Rhinosinusitis
CSF	Cerebrospinal Fluid
CW	Clockwise
CCW	Counterclockwise
DO	Doctor of Osteopathy
Ep	Epithelium
FVC	Forced Vital Capacity
FEV1	Forced Expiratory Volume in 1 Second
FVC/FEV1	Ratio of Forced Vital Capacity to Forced Expiratory Volume in 1 Second
FEF 25–75%	Forced Expiratory Flow at 25–75% of Pulmonary Volume
HFCWO	High-Frequency Chest Wall Oscillation
HLVA	High-Velocity, Low-Amplitude
HRV	Heart Rate Variability
ICS	Intercostal Space
Ig	Immunoglobulin
IL	Interleukin
ILCs	Innate Lymphoid Cells
ME	Muscle Energy
MFR	Myofascial Release
OMT	Osteopathic Manipulative Treatment
OA	Occipitoatlantal (Joint)
OA-D	Occipitoatlantal Decompression
PRM	Primary Respiratory Mechanism
PFT	Pulmonary Function Test
QoL	Quality of Life
RCT	Randomized Controlled Trial
SD	Student Doctor
Sm	Smooth Muscle
T3	Thoracic Vertebra 3
Th	T Helper (cell)
